# Extracellular Fe^2+^ and Fe^3+^ modulate osteocytic viability, expression of SOST, RANKL and FGF23, and fluid flow-induced YAP1 nuclear translocation

**DOI:** 10.1038/s41598-023-48436-3

**Published:** 2023-12-01

**Authors:** Wasutorn Chankamngoen, Saowalak Krungchanuchat, Jirawan Thongbunchoo, Naraporn Sirinonthanawech, Jarinthorn Teerapornpuntakit, Nattapon Panupinthu, Narattaphol Charoenphandhu

**Affiliations:** 1 Graduate Program in Molecular Medicine, Faculty of Science, Mahidol University, Bangkok, 10400 Thailand; 2 Center of Calcium and Bone Research (COCAB), Faculty of Science, Mahidol University, Bangkok, 10400 Thailand; 3 Department of Physiology, Faculty of Science, Mahidol University, Rama VI Road, Bangkok, 10400 Thailand; 4 Institute of Molecular Biosciences, Mahidol University, Nakhon Pathom, 73170 Thailand; 5 Department of Physiology, Faculty of Medical Science, Naresuan University, Phitsanulok, 65000 Thailand; 6 The Academy of Science, The Royal Society of Thailand, Dusit, Bangkok, 10300 Thailand

**Keywords:** Bone, Apoptosis, Endocrine system and metabolic diseases

## Abstract

Iron overload negatively affects bone mass and strength. However, the impact of iron excess on osteocytes—important bone cells for mechanotransduction and remodeling—is poorly understood. Herein, we examined the effects of iron exposure on osteocytes during their maturation process. We discovered that iron overload caused apoptosis of osteocytes in early and late stages of differentiation. Notably, the expression of key proteins for iron entry was downregulated during differentiation, suggesting that mature osteocytes were less susceptible to iron toxicity due to limited iron uptake. Furthermore, iron overload also enriched a subpopulation of mature osteocytes, as indicated by increased expression of *Dmp1*, a gene encoding protein for bone mineralization. These iron-exposed osteocytes expressed high levels of *Sost*, *Tnfsf11* and *Fgf23* transcripts. Consistently, we demonstrated that exogenous FGF23 stimulated the formation and survival of osteoclasts, suggesting its regulatory role in bone resorption. In addition, iron overload downregulated the expression of *Cx43*, a gene encoding gap junction protein in the dendritic processes, and impaired YAP1 nuclear translocation in response to fluid flow in differentiated osteocytes. It can be concluded that iron overload induces cellular adaptation in differentiating osteocytes, resulting in insensitivity to mechanical stimulation and potential disruption of the balance in bone remodeling.

## Introduction

Excessive accumulation of iron in the body, also known as iron overload, is generally caused by an imbalance in iron absorption, storage, and elimination. There are several causes of iron overload, for example, genetic disorders (e.g., thalassemia, hereditary hemochromatosis), chronic liver disease, and repeated blood transfusion^1^. When iron overload occurs, it is commonly found in the liver, heart, pancreas, and certain endocrine glands and leads to organ dysfunctions. Therefore, it is not surprising to find high extracellular iron being associated with diseases like liver cirrhosis, heart failure, and diabetes mellitus^2–4^. Skeletal tissue is another site of iron overload under these circumstances. It has been shown that an excess in extracellular iron causes bone loss and increases risk of fractures^5^. Extracellular iron—both ferrous (Fe^2+^) and ferric (Fe^3+^)—can interfere with differentiation of bone forming cells, osteoblast functions, leading to aberrant bone formation^6,7^. However, little is known about the effects of iron overload on osteocytes, the most abundant bone cells (> 90% of total bone cells) that reside within mature bone tissue.

Since osteocytes play a key role in the mechanotransduction of bone remodeling that involve both bone forming osteoblasts and bone resorbing osteoclasts, dysregulation of osteocyte differentiation and functions can compromise osteoblast and osteoclast activities^8^. Generally, osteocytes are capable of secreting humoral factors such as sclerostin (SOST) that controls bone remodeling. Study in mice showed that, deletion of gene encoding hepcidin, a protein produced primarily in the liver to control whole body iron balance, caused iron overload which induced bone loss by upregulating SOST production and increasing the ratio of receptor activator of nuclear factor κB ligand to osteoprotegerin (RANKL/OPG) the latter of which indicated bone resorption^9^. Furthermore, iron overload in mature osteocyte-like MLO-Y4 cells led to production of several soluble factors that promote osteoclastogenesis in RAW264.7, the monocyte/macrophage precursor cells^10^. Furthermore, osteocytes are the primary cells responsible for fibroblast growth factor 23 (FGF23) production. Our previous work has shown that FGF23 was more abundant in hemizygous β-globin knockout thalassemic mice compared with the wild-type littermates^11^. Whether iron overload directly stimulates FGF23 production in osteocyte remains unclear. Effects of FGF23 on osteoclastogenesis from human monocytes showed biphasic responses during the course of differentiation^12^. Furthermore, how FGF23 affects survival of mature osteoclasts is not known. Thus, further investigation on how extracellular iron alters FGF23 production as well as the action of FGF23 on osteoclast biology is warranted.

Under normal conditions, osteocytes are derived from osteoblasts that become embedded within the mineralized bone matrix. Two primary functions of osteocytes include bone mineralization and mechanotransduction. Thus, there are several transcriptional changes during the transition from osteoblasts to young and mature osteocytes. Of those, upregulation of dentin matrix protein 1 (DMP1) is a key marker of mature osteocytes that are active during bone mineralization^13^. Lack of functional DMP1 in mice as a result of genetic ablation leads to defects in mineralization as seen in rickets and osteomalacia^13^. In addition, DMP1 facilitates the formation of dendritic processes that are crucial for cell–cell communication during bone remodeling^14^. The formation of interconnected osteocyte network provides an important platform for bone mechanotransduction. Maturation of the osteocytes also involves upregulation of genes for ion transport, for example, calcium channels and connexins^15^. However, the knowledge of how iron overload affects cell–cell communication and mechanosensing capacity in osteocytes is extremely limited.

Here, we explored the impact of iron overload in osteocytes during differentiation, communication and mechanotransduction. Toxicity of iron overload was clearly evident when exposure of undifferentiated primary osteocytes and osteocyte-like cells to ferrous ammonium sulfate (FAS, a donor of Fe^2+^) or ferric ammonium citrate (FAC, a donor of Fe^3+^). Interestingly, cell death was less encountered in differentiated osteocytes. We proposed that under an iron overload condition, these differentiated cells were able to reduce iron uptake through downregulation of iron transport proteins that mediate iron entry coupled with upregulation of those that reduce intracellular iron accumulation. This was in line with the observed enrichment of mature DMP1-positive osteocytes during differentiation under iron excess condition. These mature osteocytes, however, displayed aberrant functions. Extracellular iron species upregulated expression of *Sost*, *Tnfsf11* and *Fgf23*, gene encoding key soluble proteins that could interfere with bone remodeling. Importantly, these mature osteocytes that differentiated under iron excess condition were less responsive to mechanical stimulation, thus demonstrating iron overload induced impairment of mature osteocyte function.

## Results

### Acute iron overload selectively decreased cell viability in undifferentiated osteocyte-like cells

**Figure 1:**
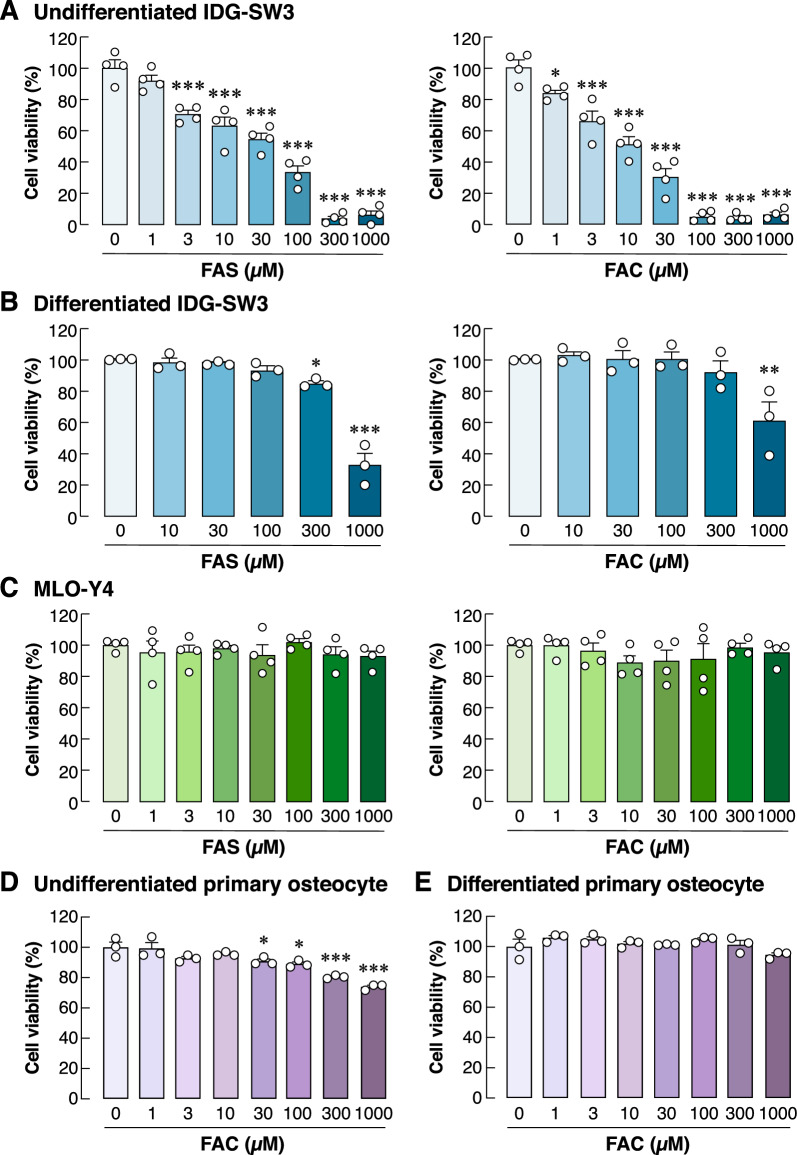
Acute exposure of extracellular iron species selectively reduced viability of undifferentiated osteocytes. We performed MTT assay in (**A**) undifferentiated, (**B**) 14-day differentiated IDG-SW3 osteocyte-like cultures, (**C**) MLO-Y4 mature osteocyte-like cultures, (D) undifferentiated primary osteocyte cultures, and (**E**) 14-day differentiated primary osteocyte cultures upon exposure to FAS or FAC at 0–1000 µM as indicated for 24 h. Percentages of cell viability were analyzed. Data are means ± SEM (*n* = 3 independent preparations in differentiated IDG-SW3, undifferentiated and differentiated primary osteocyte cultures and *n* = 4 independent preparations in undifferentiated IDG-SW3 and MLO-Y4 cultures). *p < 0.05, **p < 0.01, ***p < 0.001 compared with conditions in which FAS or FAC were absent (i.e., 0 µM), analyzed by One-way ANOVA with Bonferroni’s multiple comparison test.

We first aimed to demonstrate the acute effects of iron exposure in osteocytes. Two populations of osteocytes were tested, i.e., undifferentiated and differentiated populations. Our working hypothesis was that if we allowed the differentiation process to complete, the toxicity due to iron excess would be diminished. To test this working hypothesis, we added extracellular iron species into cultures of undifferentiated and differentiated IDG-SW3 cells for only 24 h. Cell viability was assessed using MTT assay in the presence of FAS and FAC at different concentrations ranging from 0 to 1000 µM. For undifferentiated IDG-SW3, we found that FAS and FAC reduced cell viability in a dose-dependent manner after 24-h incubation starting from 3 µM for FAS and 1 µM for FAC (Fig. 1A). The half maximal inhibitory concentration (IC_50_) at 24-h incubation for FAS was 54.4 µM and that for FAC was 14.5 µM. Importantly, viability of differentiated IDG-SW3 was unaffected by the excess of extracellular iron species up to 100 µM for FAS and 300 µM for FAC (Fig. 1B). To further demonstrate the resistance to iron excess in fully differentiated (mature) osteocytes, MLO-Y4 cells were used. Exposure to iron excess (0–1000 µM) had no significant effect on cell viability (Fig. 1C). In the subsequent step, primary osteocytes were isolated from murine long bones. FAC significantly reduced the viability of undifferentiated primary murine osteocytes (Fig. 1D), while viability of differentiated primary osteocytes remained unaffected by iron excess (Fig. 1E). Taken together, it appeared that undifferentiated osteocytes were selectively affected by acute exposure to extracellular iron species.

### Continuous exposure to extracellular iron species led to apoptosis of both differentiated and undifferentiated osteocyte-like cells

**Figure 2:**
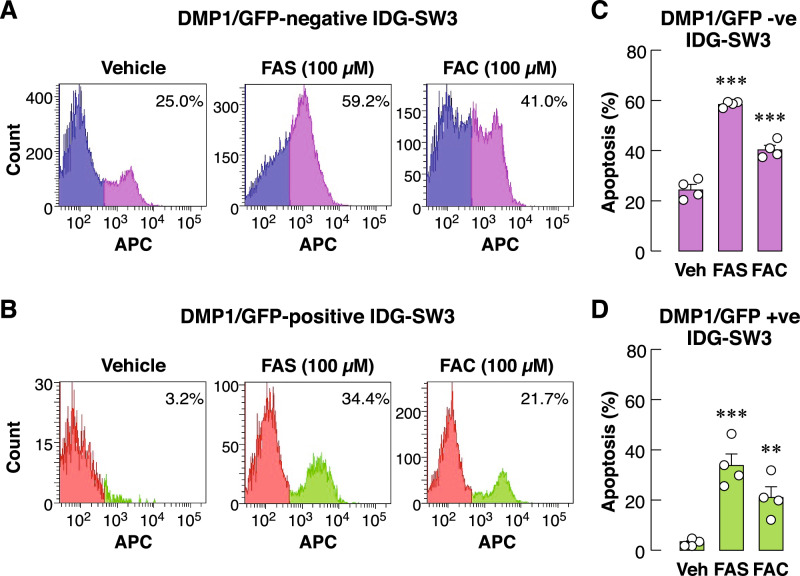
Extracellular iron species induced apoptosis of differentiated and undifferentiated IDG-SW3 osteocyte-like cells. Differentiation of IDG-SW3 were induced by supplementation of 50 µg/ml ascorbic acid and 4 mM β-glycerophosphate in the culture media for 7 days. During this period, FAS (100 µM), FAC (100 µM) or vehicle was added. These IDG-SW3 cultures contained mixed populations of cells at various stages of differentiation. DMP1/GFP signals were used to separate (**A**) undifferentiated IDG-SW3 (i.e., DMP1/GFP-negative) and (**B**) differentiated IDG-SW3 (i.e., DMP1/GFP-positive) subpopulations using flow cytometry. In each subpopulation, APC Annexin-V staining was performed. Percentages of apoptotic cells in (**C**) undifferentiated IDG-SW3 and (**D**) differentiated IDG-SW3 subpopulations were quantified. Data are means ± SEM (*n* = 4 independent preparations). ***p < 0.001 compared with vehicle-treated groups analyzed by One-way ANOVA with Bonferroni’s multiple comparison test.

Since it is known that iron overload persisted throughout the process of osteocyte differentiation in vivo, IDG-SW3 osteocyte-like cells were differentiated for 7 days in the presence of FAS or FAC at 100 µM, a concentration based on previously characterized in UMR-106 osteoblast-like cells^6^. Next, we induced differentiation of IDG-SW3 cultures yielding mixed populations of undifferentiated and differentiated cells. Thus, it would be interesting to see whether extracellular irons could induce apoptosis in both subpopulations. First, we employed another assay for assessment of cell number using flow cytometry technique to quantify propidium iodide (PI)-positive cells. Exposure to FAS or FAC (100 µM) for 7 days indeed did not altered percentages of PI-positive cells in differentiated IDG-SW3 cultures (Fig. S1). We then separated IDG-SW3 cultures into two populations based on DMP1/GFP protein levels using flow cytometry to represent undifferentiated and differentiated populations^16^. Apoptotic assays were then performed to assess the toxicity of prolonged iron overload for 7 days during differentiation. At baseline, DMP1/GFP-negative population with high APC signals was approximately 25% of the total population (Fig. 2A). Addition of extracellular iron species further induced apoptosis in this DMP1/GFP-negative population (Fig. 2C). For DMP1/GFP-positive population, we observed minimal apoptotic signals at baseline at approximately 3% (Fig. 2B). Similarly, these DMP1-positive IDG-SW3 osteocyte-like cells were sensitive to FAS and FAC (Fig. 2D). These data demonstrated that both differentiated and undifferentiated IDG-SW3 osteocyte-like cells were sensitive to prolonged iron overload. Note, the percentages of apototic cells were reduced in a subpopulation of differentiated osteocytes. Taken together, iron toxicity in osteocytes was profound in all stages of osteocyte differentiation. Prolonged exposure to either extracellular ferrous or ferric iron led to osteocyte death, in part, via apoptosis during the course of differentiation.

### Exposure to extracellular iron species led to changes in gene expression of several iron transport proteins during osteocyte differentiation

**Figure 3:**
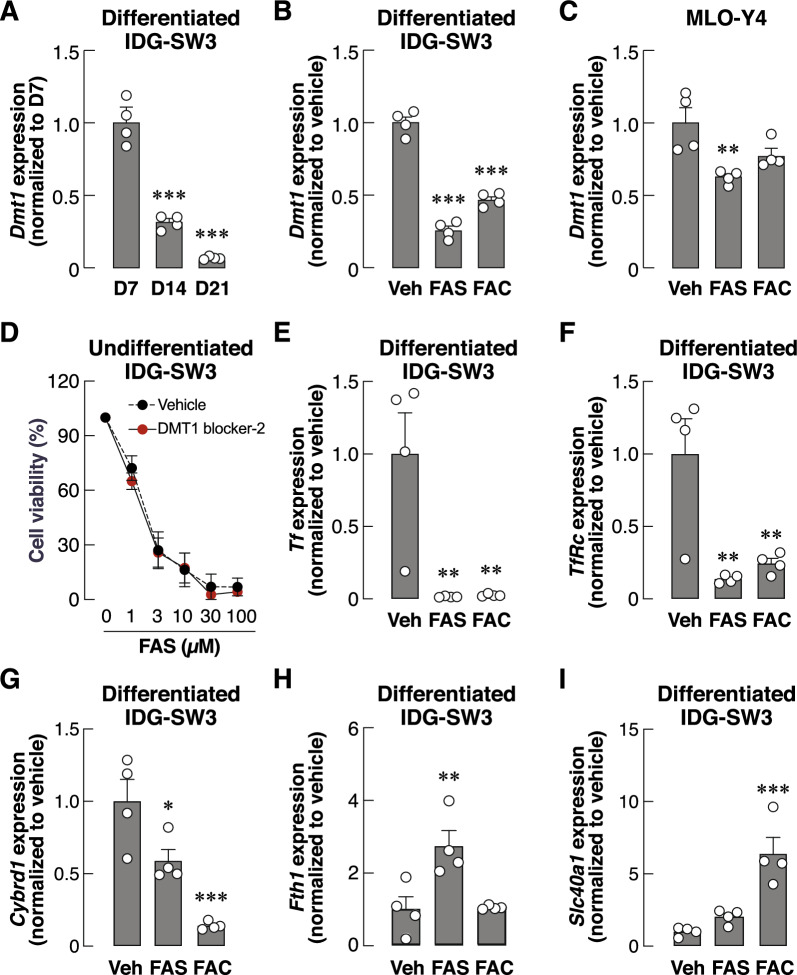
Extracellular iron species altered gene expression of several iron transport proteins in differentiated osteocyte-like cultures. IDG-SW3 osteocyte-like cultures were differentiated for 7–21 days and total RNA samples were collected for quantitative real-time RT-PCR. (**A**) Expression levels of *Dmt1* transcripts were quantified and analyzed. (**B**) In some experiments, IDG-SW3 cultures were treated with FAS (100 µM), FAC (100 µM) or vehicle for 14 days during differentiation. Expression levels of *Dmt1* transcripts were analyzed. (**C**) Expression levels of *Dmt1* transcripts were also quantified and analyzed in cultures of MLO-Y4 mature osteocyte-like cultures treated with FAS (100 µM), FAC (100 µM) or vehicle for 7 days. (**D**) MTT assays were performed in undifferentiated IDG-SW3 cells under FAS exposure at 0–100 µM in the presence of DMT1 blocker-2 (0.3 µM) or vehicle. Quantitative RT-PCR were performed using mRNA samples from 14-day differentiated IDG-SW3 for expression of gene encoding (**E**) transferrin, (**F**) TfR1, (**G**) CYBRD1, (**H**) ferritin, and (**I**) FPN1. Data are means ± SEM (*n* = 4 independent preparations). **p < 0.01, ***p < 0.001 compared with vehicle-treated groups analyzed by One-way ANOVA with Bonferroni’s multiple comparison test.

It was interesting that the majority of differentiated IDG-SW3 cells did not undergo apoptosis when exposed to FAS or FAC (Fig. 2C, red-labelled populations). This was consistent with the continuous presence of mature osteocytes resisting iron overload condition in vivo^9^. We thus hypothesized that these mature cells had acquired a mechanism by which iron entry was restricted or prevented. It is well characterized that DMT1 is a primary entry point of free extracellular iron, particularly Fe^2+^, in many cell types^17^. Thus, we examined changes in *Dmt1* mRNA levels during the course of osteocyte differentiation using quantitative real-time reverse-transcription polymerase chain reaction (qRT-PCR). The levels of *Dmt1* mRNA were markedly reduced between day 7 and day 21 of differentiation (approximately 5.29 folds within 2 weeks) (Fig. 3A). Interestingly, extracellular iron species in excess (i.e., FAS and FAC at 100 µM) further induced a significant reduction in *Dmt1* mRNA levels in differentiated IDG-SW3 osteocyte-like cells at day 14 (Fig. 3B). We also observed the similar effect of extracellular FAS on *Dmt1* mRNA expression in mature MLO-Y4 osteocyte-like cells (Fig. 3C).

We further postulated that the iron-induced decrease in *Dmt1* expression during osteocyte differentiation probably helped protecting the cells from iron toxicity. Next, we asked whether the blockade of DMT1 could enhance cell viability upon exposure to extracellular irons. DMT1 blocker-2, which was found to inhibit intestinal iron uptake^18^, was used. Surprisingly, we found that DMT1 blocker-2 alone reduced viability of undifferentiated IDG-SW3 cells in a dose-dependent manner (Fig. S2, IC_50_ = 1.54 µM). This finding suggested that inhibition of DMT1 severely disrupted the transport mechanisms of several divalent cations and cellular metabolism. Importantly, there was no significance changes in the effects of FAS-induced reduction in viability of undifferentiated IDG-SW3 cells in the presence of DMT1 blocker-2 (0.3 µM, a concentration that did not affect cell viability itself) compared to vehicle (Fig. 3D).

The ineffectiveness of DMT1 blockade as a target to promote osteocyte survival prompted us to further explore additional players in iron transport in osteocytes, i.e., Fe^3+^ entry by transferrin and transferrin receptor (TfR1), Fe^3+^-to-Fe^2+^ conversion by cytochrome B reductase (CYBRD1), intracellular binding of Fe^2+^ by ferritin and Fe^2+^ release by ferroportin 1 (FPN1). Using qRT-PCR, we found that FAS and FAC drastically reduced expression of genes encoding transferrin and TfR1 (Fig. 3E,F). Expression of gene encoding CYBRD1 also significantly decreased upon exposure of FAS or FAC (Fig. 3G). On the other hand, these cells upregulated the expression of gene encoding ferritin (Fig. 3H) and FPN1 (Fig. 3I) upon FAS and FAC exposure, respectively. Taken together, these data indicated that differentiating osteocytes actively respond to iron overload. Prevention of osteocyte death caused by iron overload is therefore challenging due to the dynamic alterations of these iron transport proteins.

### Iron overload enhanced osteocyte differentiation and expression of proteins that control bone remodeling

**Figure 4:**
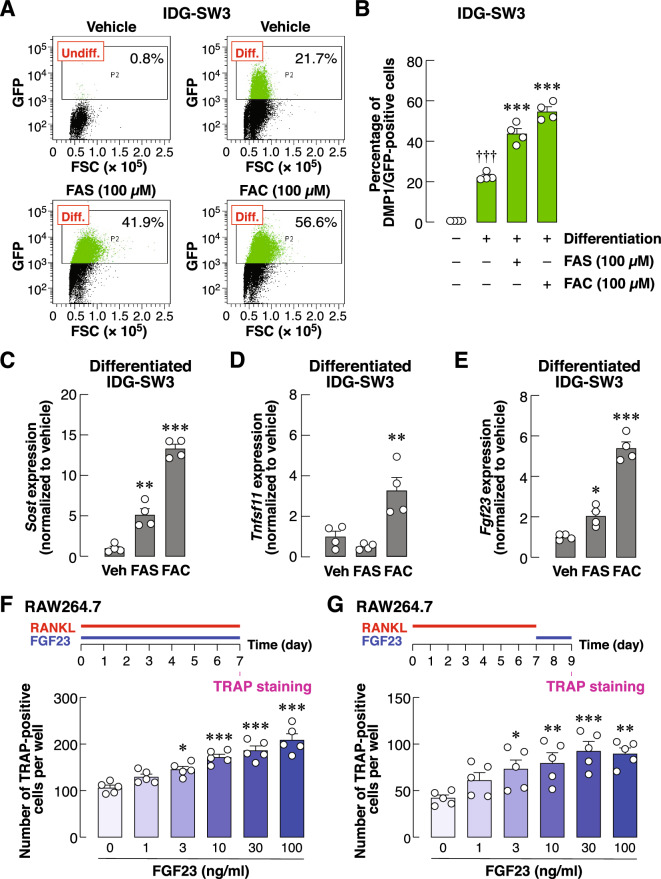
Extracellular iron species promoted transcript and protein expression of mature osteocyte-like cells. (**A**) IDG-SW3 osteocyte-like cultures were differentiated for 14 days in the presence of FAS (100 µM), FAC (100 µM) or vehicle. Cells were harvested and expression of DMP1/GFP fusion proteins were quantified using flow cytometry. Representative populations and percentages of DMP/GFP-positive osteocyte-like cells in each condition were shown. (**B**) All data from the flow-cytometry studies were quantified. In some experiments, total RNA samples were collected from differentiated IDG-SW3 cultures treated with FAS (100 µM), FAC (100 µM) or vehicle for 14 days. Quantitative RT-PCR analyses were performed using specific primers for (**C**) *Sost*, (**D**) *Tnfsf11* (**E**) *Fgf23*. Data were quantified and normalized to vehicle-treated groups. (**F**) RAW264.7 cells were induced to differentiate into multinucleated osteoclast-like cells in the presence of RANKL (50 ng/ml) and FGF23 (0–100 ng/ml) for 7 days. (**G**) In some experiments, RAW264.7 cells were induced to differentiate into multinucleated osteoclast-like cells in the presence of RANKL (50 ng/ml) alone for 7 days. FGF23 (0–100 ng/ml) were then added into these culture for another 2 days. TRAP staining was performed, and numbers of TRAP-positive cells were quantified. Data are means ± SEM (*n* = 4 independent preparations for IDG-SW3 studies and *n* = 5 independent preparations for RAW264.7 studies). ^†††^*p* < 0.001 compared with undifferentiated IDG-SW3 cultures, analyzed by unpaired Student’s *t*-test. *p < 0.05, **p < 0.01, ***p < 0.001 compared with vehicle-treated groups or conditions in which FGF23 was absent (i.e., 0 ng/ml), analyzed by One-way ANOVA with Bonferroni’s multiple comparison test.

The selective actions of extracellular iron on mature osteocytes, i.e., alteration of gene expression and resistance to iron overload, led us to question whether iron overload directly altered osteocyte differentiation. Levels of DMP1 expression were again used to assess osteocyte differentiation using GFP intensities. There was significant increase in DMP1/GFP-positive cell populations in differentiated IDG-SW3 cultures (Fig. 4A,B, vehicle-treated panels). Interestingly, exposure to extracellular iron species further enhanced the GFP signals in the differentiated cultures (Fig. 4A,B, FAS and FAC-treated panels). Moreover, we found that addition of extracellular iron species significantly upregulated levels of DMP1 transcripts in IDG-SW3 culture under differentiating condition (Fig. S3). These data thus indicated that extracellular iron species enhanced DMP1 expression during osteocyte differentiation.

Next, we investigated possible outcomes from iron-induced osteocyte differentiation. SOST, RANKL and FGF23—the osteocyte-derived humoral factors—are exclusive markers of functional osteocytes that regulate calcium and bone metabolism^19–21^. Indeed, we found that exposure to extracellular iron species markedly induced the expression of *Sost* mRNA (i.e., 5.15-fold for FAS and 13.34-fold for FAC) in differentiated IDG-SW3 osteocyte-like cells (Fig. 4C). Similarly, extracellular iron species significantly upregulated the expression of *Tnfsf11* mRNA (i.e., 3.28-fold for FAC, Fig. 4D) and *Fgf23* mRNA (i.e., 2.03-fold for FAS and 5.39-fold for FAC, Fig. 4E) in these cells.

Functions of SOST in bone remodeling process are well characterized, i.e., it antagonizes Wnt-induced bone formation. In addition, roles of RANKL on osteoclastogenesis and bone resorption are well established. However, little is known regarding the role of FGF23, especially its action on osteoclasts. Previously, it has been shown that conditioned media of osteocyte culture exposed to extracellular iron stimulated differentiation of RAW264.7-derived osteoclasts^10^. Since the conditioned media contained a variety of active molecules that modulated osteoclast differentiation, we thus examined the possible effect of FGF23 in this process using recombinant FGF23. First, we added FGF23 during the period of differentiation driven by RANKL and monitored the number of TRAP-positive cells at day 7. FGF23 further increased formation of RAW264.7-derived osteoclasts in a dose-dependent manner (Fig. 4F). Secondly, we explored the effect of FGF23 on osteoclast survival after the 7-day period of RANKL-induced osteoclast formation. After differentiation, the presence of FGF23 in cultures for an additional 2 days promoted cell survival starting at 3 ng/ml and reaching maximum at 30 ng/ml (Fig. 4G). These findings suggest that iron overload stimulates osteocyte maturation and likely modulates functions of other bone cells (e.g., osteoclasts) via iron-induced production of key soluble factors (e.g., RANKL and FGF23).

### Exposure to extracellular iron species impaired response of osteocytes to mechanical stimuli

**Figure 5:**
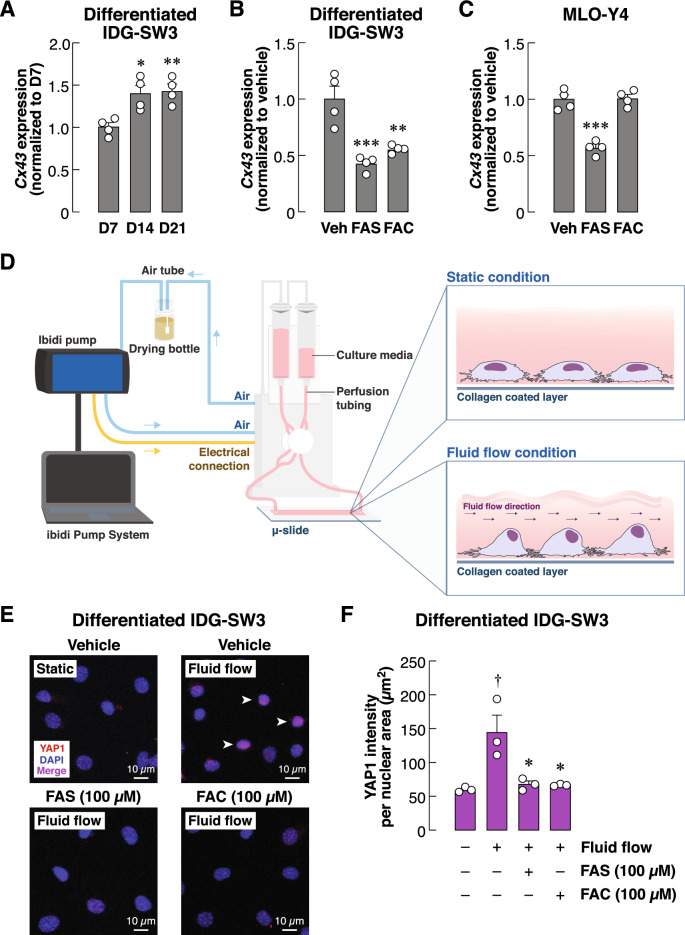
Extracellular iron species decreased *Cx43* transcript levels and suppressed flow-induced YAP1 nuclear translocation in mature osteocyte-like cultures. (**A**) IDG-SW3 osteocyte-like cultures were differentiated for 7–21 days. (**B**) IDG-SW3 cultures were induced to differentiate for 14 days in the presence of FAS (100 µM), FAC (100 µM) or vehicle. (**C**) Mature MLO-Y4 cultures were treated with FAS (100 µM), FAC (100 µM) or vehicle for 7 days. Total RNA samples were collected to assess *Cx43* mRNA expression. Expression levels of *Cx43* transcripts were analyzed. In other experiments, IDG-SW3 osteocyte-like cultures were differentiated for 14 days. Cultures were then exposed to FAS (100 µM), FAC (100 µM) or vehicle for another 24 h. (**D**) Continuous fluid flow at 2 Pa was then applied to these osteocyte-like cultures. In some experiments, vehicle-treated IDG-SW3 cultures were left under static condition. (**E**) Nuclear translocation of YAP1 were assessed using immunofluorescence technique and visualized by confocal microscopy. (**F**) YAP1 intensity normalized by nuclear area were quantified. Data are means ± SEM (*n* = 4 independent preparations for quantitative RT-PCR studies and *n* = 3 independent preparations for fluid flow studies). ^†^*p* < 0.05 compared with static condition, analyzed by unpaired Student’s *t*-test. *p < 0.05, **p < 0.01, ***p < 0.001 compared with vehicle-treated groups, analyzed by One-way ANOVA with Bonferroni’s multiple comparison test.

Having demonstrated the potential effects of iron overload on communication of bone cells initiated by osteocytes, we next examined the primary intrinsic functions of osteocytes, i.e., sensing and transducing mechanical stimuli in bone. Cx43 is the main gap junction protein used for communication between neighboring mature osteocytes during these events^15^. In accord, we showed that the expression of *Cx43* mRNA was upregulated in IDG-SW3 osteocyte-like cells during differentiation (Fig. 5A). Interestingly, extracellular iron species present throughout the 14-day differentiation significantly reduced the levels of Cx43 expression by approximately 50% (Fig. 5B). Moreover, suppression of Cx43 expression was also observed in mature osteocyte-like MLO-Y4 cells by ~ 50% after being treated with FAS (Fig. 5C). Nevertheless, we did not observe this inhibitory effect with FAC in MLO-Y4.

Downregulation of *Cx43* in differentiated IDG-SW3 cultures after exposure to extracellular iron suggested that response of these cells to mechanical stimuli was impaired. To evaluate this hypothesis, we assessed nuclear translocation of *yes* associated protein 1 (YAP1)—a proxy indicator of cellular mechanotransduction—using fluid-flow stimulation (the ibidi Pump System, Fig. 5D) coupled with immunofluorescence. We found that non-stimulated IDG-SW3 cells under static condition had minimal signals of YAP1 in nuclei (Fig. 5E,F). Under fluid-flow stimulation, nuclear translocation of YAP1 was drastically increased. Importantly, 24-h pretreatment of FAS and FAC significantly decreased flow-induced YAP1 nuclear translocation in these cells (Fig. 5F). These data clearly indicated that exposure to extracellular iron species impaired YAP1-mediated mechanotransduction pathway in these differentiated IDG-SW3 osteocyte-like cells.

## Discussion

Osteocytes are the most abundant cell type in the skeleton. These cells are long-lived and situated within the lacunar network filled with extracellular fluid. Thus, any changes in the constituents of this fluid, for example, rising extracellular iron levels, can lead to prolonged and extended adverse effects on the fate and function of these cells. Indeed, several lines of evidence indicated that iron overload induced osteocyte death by apoptosis^9^. This phenomenon was in line with our previous findings in osteoblasts, the precursors of osteocytes, when exposed to extracellular iron^17^. Interestingly, extracellular iron could also interfere with the osteogenic potentials of human bone-marrow mesenchymal stem cells^22^. This profound toxicity of extracellular iron ultimately led to decreases in bone mass and bone strength^23^. However, it remained a puzzle that certain population of osteocytes continued to thrive in the living bones under iron excess. Here, we uncovered the discrepancy of iron toxicity among subpopulations of osteocytes. We found that undifferentiated/immature osteocytes were highly susceptible to iron overload. In contrast, differentiated/mature osteocytes were resilient under iron excess. We further propose here that fully differentiated (mature) osteocytes are able to withstand prolonged iron exposure by limiting the entry and promoting the disposal of intracellular sources. Taken together, our data suggest that there is a reversal of iron susceptibility during osteocyte maturation.

It is known that Fe^2+^ is the form of free iron that can be transported directly into several cell types^24^. Epithelial cells of duodenum express cytochrome B reductase 1 (CYBRD1), namely duodenal cytochrome B (DcytB), which is used for Fe^3+^-to-Fe^2+^ conversion during intestinal iron absorption^24^. It is conceivable that CYBRD1 may be attractive for targeted therapy that would prevent the Fe^3+^-to-Fe^2+^ conversion and limit iron entry into osteocytes and perhaps other bone cells. Interestingly, we herein found that expression levels of CYBRD1/DMT as well as transferrin/TfR1 systems were decreased by FAS and FAC in differentiated osteocytes. Downregulation of these iron entry pathways in mature osteocytes could be the key limiting steps that need to overcome for improving osteocyte functions and bone health. At cellular levels, chronic exposure to iron caused intracellular iron overload and accumulation of iron-containing vesicles derived from lysosomes^25^. Excess intracellular iron then induced generation of reactive oxygen species that interfere with a variety of cellular functions^25^. This concept of iron-containing vesicle formation and accumulation has been implicated as one of the consequences of neuronal damages in neurogenerative diseases, such as Alzheimer’s and Parkinson’s diseases^26^.

It has been shown that DMP1 expression exerted protective role in osteocytes under hyperphosphatemia^27^. Here, we similarily found that DMP-positive population in differentiated IDG-SW3 cultures exhibited minimal percentages of apoptotic cells. Moreover, we found that expression of *Dmp1* mRNA and DMP1/GFP fusion protein were increased in differentiating IDG-SW3 cultures upon iron exposure. However, we did not find that upregulation of DMP1 expression could protect osteocytes under iron overload. Our data highlight that the protective actions of DMP1 were selective and depended on certain changes in bone microenvironment. How osteocytes resist iron overload and survive as well as the underlying mechanism are subject for further investigation. In this regard, metallothionein may represent a putative molecule that could mitigate iron toxicity in osteocytes since it has been identified as metal-binding protein that prevented cadmium toxicity^28^. Iron-induced enrichment of DMP1-positive cells in differentiating osteocyte culture also required further explanation. It was possible that upregulation of DMP1 under iron-overload conditions was a result of selective susceptibility of undifferentiated/immature osteocytes to iron overload. Alternatively, it was possible that extracellular iron species exerted direct actions on the pathways that upregulated *Dmp1* mRNA expression. In this regard, limited studies have identified Notch signaling pathway, activation of JunB and intrinsic histone acetyltransferase activity of p300, as key mediators of DMP1 expression during osteocyte differentiation^29,30^.

Functions of DMP1 in osteocytes are involved with dendritic formation and bone mineralization^14^. For instance, knockout of *Dmp1* in rabbits caused defects in the structure of Haversian canals and formation of bone matrix^31^. We, however, postulated that these DMP1-mediated functions did not properly manifest under iron overload. First, our data indicated that expression of *Cx43* mRNA was downregulated under iron excess. Since Cx43 is a predominant gap-junction protein found on dendritic processes of functional osteocytes^32^, it is possible that the osteocyte dendrites formed under iron excess are functionally incompetent. Indeed, we found that extracellular iron species diminished fluid-flow-induced YAP1 nuclear translocation. Therefore, our work highlighted a marked decrease in mechanosensitivity of osteocytes under iron overload. Moreover, generation of oxidative stress has been implicated in inhibitory effects of iron in the production of prostaglandin E_2_ and nitric oxide^33,34^. Therefore, it is tempting to speculate that accumulation of intracellular iron induces the loss of mechanosensitivity in osteocytes by inhibiting the synthesis of these two molecules.

We found that DMP1-enriched osteocyte cultures expressed elevated levels of *Sost* mRNA under iron excess. Since SOST produced by mature osteocytes is known to inhibit osteogenesis via suppression of Wnt/β-catenin pathway during osteoblast differentiation^35^, we proposed here that iron overload reduced bone mass, in part, via osteocyte-mediated increase in SOST production that interfered with osteoblastogenesis. Consistently, conditioned medium of MLO-Y4 cells treated with FAC was found to suppress osteogenic potentials in MC3T3-E1 osteoblastic cultures^9^. Here, we demonstrated that expression of *Tnfsf11* and *Fgf23* mRNA transcripts was upregulated in DMP1-enriched osteocyte cultures. In addition, we postulated that FGF23 could promote differentiation and survival of osteoclast-like cells derived from RAW264.7 macrophages. In this regard, FGF23 was reported to stimulate bone resorptive activity in cells derived from human monocytes^12^. Moreover, serum concentrations of C-terminal telopeptide of collagen I (CTX), a marker of bone resorption were elevated in hypophosphatemic transgenic mice overexpressing FGF23^36^. Taken together, mature osteocytes developed under iron excess were functionally defective, i.e., insensitive to fluid-flow stimulation, and were likely to disrupt bone remodeling via increased production of SOST, RANKL and FGF23.

Impairment of osteocyte function and aberrant FGF23 production should also be emphasized in iron deficiency, another spectrum of iron dyshomeostasis. In this regard, lack of extracellular iron in bone disrupted osteocyte maturation from osteoblasts isolated from patients with chronic kidney disease^37^. This finding was in line with our current finding in which iron excess accelerated osteocyte maturation. However, the early differentiated osteocytes under iron deficiency unexpectedly expressed high amounts of FGF23. Increased FGF23 production in iron deficiency has also been described in patients with X-linked hypophosphatemic rickets^38^. Under this condition, periosteocytic lesions and mineralized defects had also been detected. Taken together, it is critical to regulate iron levels within bone microenvironment for proper osteocyte functions and appropriate FGF23 production.

Taken together, our results described defects in osteocyte functions that were consistent with skeletal phenotypes found in iron overload condition. Therefore, we propose that interventions that can prevent iron overload and/or limit the actions of extracellular irons in osteocyte should provide therapeutic benefits. To date, iron chelation is a primary method for preventing iron overload in clinic. Additional benefits of iron chelation on improving bone mass and strength have also been implicated, for example, the use of deferoxamine in mice lacking hepcidin^9^. Nevertheless, our current findings cautioned that targeting individual protein that involved in iron transport might be toxic and ineffective (i.e., the use of DMT1 blocker-2 to prevent iron toxicity). In addition, blockade of DMT1 actions may result in deficiencies of several trace elements, which can also lead to bone loss^39^.

Alternatively, it is possible to restore bone health under iron overload by limiting the negative actions of SOST, a known suppressorof Wnt-induced bone formation^40^ as well as RANKL and FGF23, mediators for osteoclast formation and survival (Fig. 4C–E), respectively. In this regard, romosozumab, a neutralizing monoclonal antibody for SOST, showed protective effects on postmenopausal bone fracture as demonstrated by the upregulation of osteoblast markers^41,42^. Neutralizing of RANKL using denosumab has been implemented in prevention of vertebral, non-vertebral and hip fractures in postmenopausal women with osteoporosis^43^. Likewise, emerging evidence implicated that the use of FGF23 neutralizing antibody improved mineralization in mouse model of hypophosphatemic rickets/osteomalacia^44^. These antibodies should be attractive tools for correcting skeletal defects under iron excess.

Based on these current findings, we reason that restoration of mechanosensitivity in mature osteocytes under iron overload is highly critical and yet challenging. Identification of iron-sensitive mechanosensors in osteocytes is requisite. The list of candidates includes ion channels, primary cilium, integrins and connexins^45^. In addition, signaling pathways surrounding YAP/TAZ in response to mechanical stimuli in these cells should also be investigated.

## Materials and methods

### Osteocyte-like cell culture and differentiation

IDG-SW3 cells were procured from Kerafast, Inc. (Boston, MA, USA). These cells, identified as undifferentiated osteocyte-like cells, were maintained in a proliferative state in T25 flasks (Corning Inc., Corning, NY, USA) pre-coated with rat-tail type I collagen (0.15 mg/ml, Sigma-Aldrich, St. Louis, MO, USA) at 4 °C overnight. The proliferation medium consisted of α-MEM medium (Gibco-Life Technologies Corporation, Grand Island, NY, USA) supplemented with 10% fetal bovine serum (FBS, Sigma-Aldrich, St. Louis, MO, USA), 100 U/ml penicillin–streptomycin (Gibco-Life Technologies Corporation, Grand Island, NY, USA), and 50 U/ml recombinant mouse interferon-γ (INF-γ, Gibco-Life Technologies Corporation, Grand Island, NY, USA) at 33 °C with 5% CO_2_. Subsequently, for further experiments, IDG-SW3 cells were seeded on rat-tail type I collagen-coated multi-well plates, dishes, or chamber slides and cultured at 37 °C with 5% CO_2_. To maintain an undifferentiated state, proliferation medium with recombinant mouse INF-γ was used. To induce differentiation, proliferation medium without recombinant mouse INF-γ was supplemented with 50 µg/ml ascorbic acid and 4 mM β-glycerophosphate (Sigma-Aldrich, St. Louis, MO, USA). In all conditions, the media were changed every 3 days.

MLO-Y4 cells were also obtained from Kerafast, Inc. (Boston, MA, USA). These cells, characterized by mature osteocyte features similar to primary cells, displayed the manifestation of an outgrowth osteocyte phenotype with strong expression of E11/GP38^46^. Given their mature osteocyte characteristics, these cells were maintained in a differentiated state in rat-tail type I collagen-coated flasks, using α-MEM medium supplemented with 5% FBS, 5% calf serum (Sigma-Aldrich, St. Louis, MO, USA), and 100 U/ml penicillin–streptomycin at 37 °C with 5% CO_2_. For subsequent experiments, MLO-Y4 cells were cultured under the same conditions in rat-tail type I collagen-coated multi-well plates or dishes. Media changes were performed every 3 days.

### Primary osteocyte culture and differentiation

Six-week-old male C57BL/6 mice were obtained from Nomura Siam International (Bangkok, Thailand). During a 3-day acclimatization period, the animals were housed in polycarbonate shoebox cages in a controlled environment maintained at 21 ± 1 °C, with a 12-h light–dark cycle and 50–60% relative humidity. Daily health assessments were conducted by veterinarians at the Central Animal Facility, Faculty of Science, Mahidol University (MUSC-CAF)—an AAALAC (Association for Assessment and Accreditation of Laboratory Animal Care)-accredited facility. Standard laboratory food and reverse-osmosis water were provided ad libitum. Primary osteocytes were isolated from the long bones of these mice using a previously described protocol that employed the alternate collagenase/EDTA digestion method^46^. Bone pieces from three mice were pooled in each cell preparation. The isolated osteocytes were then cultured on type-I rat tail collagen-coated flasks with proliferation medium, excluding recombinant mouse INF-γ, for subsequent experiments. To induce differentiation, the proliferation medium, again without recombinant mouse INF-γ, was supplemented with 50 µg/ml ascorbic acid and 4 mM β-glycerophosphate. The media were changed every 3 days. Primary osteocyte cultures were validated by morphological examination and the expression of markers, e.g., *Sost*, *Fgf23* and *Cx43* transcripts. This protocol has been approved by the Institutional Animal Care and Use Committee (IACUC), Faculty of Science, Mahidol University, and the work was conducted in accordance with relevant guidelines and regulations, including the ARRIVE guideline (https://arriveguidelines.org).

### Cell viability assay

Undifferentiated and 14-day differentiated IDG-SW3 cells, as well as MLO-Y4 cells, were seeded in 96-well plates at a density of 3,000 cells/well and cultured with α-MEM medium supplemented with 10% FBS and 100 U/ml penicillin–streptomycin for 24 h. For primary osteocyte cultures, both undifferentiated and 14-day differentiated cells were seeded in 96-well plates at a density of 8,500 cells/well and cultured with α-MEM medium supplemented with 10% FBS and 100 U/ml penicillin–streptomycin for 72 h. Subsequently, cells were treated with FAS or FAC at concentrations ranging from 0–1,000 µM for an additional 24 h. Following iron exposure, cells were incubated with 3-(4,5-dimethylthiazol-2-yl)-2,5-diphenyltetrazolium bromide (MTT) solution (Sigma-Aldrich, St. Louis, MO, USA) at a final concentration of 0.5 mg/ml and 37 °C for 4 h. The water-insoluble formazan crystals were dissolved with dimethyl sulfoxide (Merck Millipore, Burlington, MA, USA) to yield purple solutions. Colorimetric measurements were then determined by 540-nm absorbance using a microplate spectrophotometer (Metertech, Taipei, Taiwan). Each condition was performed in duplicate.

### Flow cytometry

Flow cytometry analyses were employed on IDG-SW3 cells for three specific purposes: to assess cell differentiation using DMP1/GFP signals, to determine apoptotic cell death, and to determine the actual cell count upon iron exposure. IDG-SW3 cultures were induced to undergo differentiation for either 7 or 14 days in the presence of FAS or FAC (Sigma-Aldrich, St. Louis, MO, USA), or vehicle. Following induction, cells were trypsinized using a 0.25% trypsin solution (Sigma-Aldrich, St. Louis, MO, USA) at 37 °C with 5% CO_2_ for five minutes. Detached cells were then washed and resuspended in phosphate-buffered saline (PBS). Endogenous GFP signals were determined using the BD FACS Canto flow cytometer (BD Biosciences, San Jose, CA, USA). In some experiments, detached cells were washed and resuspended in binding buffer containing APC Annexin-V or propidium iodide according to the manufacturer’s protocol (BioLegend, San Diego, CA, USA). APC signals were then quantified in both subpopulations of IDG-SW3 cells, i.e., differentiated and undifferentiated cells, using the BD FACS Canto flow cytometer (BD Biosciences, San Jose, CA, USA). Quantitative analyses were performed using BD FACS Diva software (BD Biosciences, San Jose, CA, USA).

### Quantitative reverse transcription-polymerase chain reaction (qRT-PCR)

Relative gene expression was determined using qRT-PCR. Total RNA from both differentiated and undifferentiated IDG-SW3 and MLO-Y4 cells was extracted using TRIzol reagent, following the manufacturer's protocol (Invitrogen Life Technologies Corporation, Carlsbad, CA, USA). The quality of total RNA was assessed by the 260/280 ratio using NanoDrop 2000 (Thermo Fisher Scientific Inc., Waltham, MA, USA), with a cut-off value set at 1.8. Complementary DNA (cDNA) synthesis was carried out using the iScript Select cDNA Synthesis Kit (Bio-Rad Laboratories, Hercules, CA, USA). Quantitative RT-PCR reactions were assembled using cDNA, reagents from SsoFast EvaGreen Supermix (Bio-Rad Laboratories, Hercules, CA, USA), and specific primer pairs (Table S1). PCR reactions were conducted using the QuantStudio 3 Real-Time PCR System (Applied Biosystems, Waltham, MA, USA), with annealing temperatures for all primer pairs set at 57 °C. The expression of each gene was assessed in duplicates and calculated as the mean cycle threshold (C_t_) divided by the mean C_t_ of 18s ribosomal RNA.

### Tartrate-resistant acid phosphatase (TRAP) staining

RAW 264.7 cells were procured from the American Type Culture Collection (Manassas, VA, USA). Our culture method for RAW264.7 cells was adapted from previously standardized procedures^47,48^ to yield TRAP-positive multinucleated cells capable of bone slice resorption, as determined by the release of type I collagen cross-linked C-telopeptide. In brief, cells were maintained in T25 flasks with DMEM (Sigma-Aldrich, St. Louis, MO, USA) supplemented with 10% FBS and 100 U/ml penicillin–streptomycin at 37 °C with 5% CO_2_. To induce the formation of multinucleated osteoclast-like cells, RAW 264.7 cells were cultured in differentiation medium, consisting of DMEM supplemented with 10% FBS, 100 U/ml penicillin–streptomycin, and 50 ng/ml RANKL, at 37 °C with 5% CO_2_ for 7 days. In some experiments, FGF23 (0–100 nM, R&D Systems, Minneapolis, MN, USA) was added throughout the 7-day differentiation to assess cell differentiation. In another set of experiments, FGF23 (0–100 nM) was added for an additional 2 days after the 7-day differentiation to evaluate cell survival. TRAP staining was employed to identify osteoclast-like cells in cultures using the TRAP Staining Kit (Cosmo Bio USA, Carlsbad, CA, USA). Briefly, cells were washed with PBS and fixed with a 4% paraformaldehyde solution. The fixed cells were then stained with a chromogenic substrate to demonstrate the activity of tartrate-resistant acid phosphatase. Total TRAP-positive multinucleated cells (three or more) were counted under a light microscope.

### Fluid flow stimulation and confocal microscopy

Undifferentiated IDG-SW3 cells were plated into µ-slide chambers (ibidi GmbH, Gräfelfing, Germany) and cultured in differentiation medium containing 50 µg/ml ascorbic acid and 4 mM β-glycerophosphate for 14 days. Subsequently, differentiated IDG-SW3 cells were incubated with 100 µM FAS or FAC for an additional 24 h. The treated cells were then subjected to a continuous fluid flow of 2 Pa, generated by the ibidi Pump System (ibidi GmbH, Gräfelfing, Germany), for 2 h. Following treatment, cells were fixed with a 4% paraformaldehyde solution for 10 min and permeabilized with a 0.1% Triton X-100 buffer for 15 min. Samples were blocked with a 2% BSA solution (Sigma-Aldrich, St. Louis, MO, USA) for 1 h and then incubated with a rabbit polyclonal YAP1 antibody (Cat no. PA5-87568, Invitrogen Life Technologies Corporation, Carlsbad, CA, USA) at a 1:100 ratio overnight. The secondary antibody used was goat anti-rabbit IgG Alexa Fluor 532 (Cat no. A-11009, Invitrogen Life Technologies Corporation, Carlsbad, CA, USA), which was incubated with the samples at a 1:500 ratio for 45 min. Samples were mounted and stained with DAPI using ibidi Mounting Medium with DAPI (ibidi GmbH, Gräfelfing, Germany). Fluorescence signals were captured using the Carl Zeiss LSM800 Super-Resolution Laser Scanning Confocal Microscope (ZEISS, Oberkochen, Germany). YAP1 intensities in the nuclei were quantified using ZEN Microscopy Software (Blue Edition 2.3, ZEISS, Oberkochen, Germany).

### Statistical analysis

Results were expressed as means ± standard error of the mean (SEM). Differences between two groups were analyzed using an unpaired Student’s t-test. Differences among three or more groups were assessed by one-way analysis of variance (ANOVA) followed by Bonferroni’s multiple comparison test. Statistical differences were considered significant at *p* < 0.05, as analyzed by GraphPad Prism 9 (GraphPad Software, Boston, MA, USA).

### Supplementary Information

Supplementary Figure S1.

Supplementary Figure S2.

Supplementary Figure S3.

Supplementary Table S1.

## Data Availability

The datasets used and/or analysed during the current study available from the corresponding author on reasonable request.
