# Supplementary material for: Extracellular Fe2+ and Fe3+ modulate osteocytic viability, expression of SOST, RANKL and FGF23, and fluid flow-induced YAP1 nuclear translocation
Source: Sci Rep. 2023 Dec 1;13:21173. doi: 10.1038/s41598-023-48436-3 (PMC10692318; doi:10.1038/s41598-023-48436-3)
Supplement: Supplementary file 4 — Supplementary Table S1. [file 41598_2023_48436_MOESM4_ESM.docx]

**Table S1:** Sequences of forward and reverse primers used for quantitative real-time RT-PCR

| **Gene** | **Primer** | **Sequence (5´ to 3´)** |
| --- | --- | --- |
| *Cx43* | Forward | TGGGATTGAAGAACACGGCA |
|  | Reverse | CAGGTGTAGACCGCACTCAG |
| *Cybrd1* | Forward | ATGTACAGCCTGCACAGC |
|  | Reverse | TGTCACTCCCATGAGAACC |
| *Dmp1* | Forward | AACAGTGCCCAAGATACCCC |
|  | Reverse | TCGATCGCTCCTGGTACTCT |
| *Dmt1* | Forward | TCAGAGCTCCACCATGACTG |
|  | Reverse | TGTGAACGTGAGGATGGGTA |
| *Fgf23* | Forward | CCATCAGACCATCTACAGTGCC |
|  | Reverse | ATAGCCATTCTCCAGCGTCC |
| *Fth1* | Forward | AAGTGCGCCAGAACTACCAC |
|  | Reverse | TGGTTCTGCAGCTTCATCAG |
| *Slc40a1* | Forward | CTACCATTAGAAGGATTGACCAGCTA |
|  | Reverse | ACTGGAGAACCAAATGTCATAATCTG |
| *Sost* | Forward | GCCGGACCTATACAGGACAA |
|  | Reverse | CACGTAGCCCAACATCACAC |
| *Tf* | Forward | GCGCATTCAAGTGTCTGAAA |
|  | Reverse | GAGCCACAACAGCATGAGAA |
| *TfRc* | Forward | TCGCTTATATTGGGCAGACC |
|  | Reverse | TGACCAATGCTGCTTTTCAC |
| *Tnfsf11* | Forward | CGCTCTGTTCCTGTACTTTCG |
|  | Reverse | CTCTCCAGAGTCGAGTCCTGC |
| *18S rRNA* | Forward | GTAACCCGTTGAACCCCATT |
|  | Reverse | CCATCCAATCGGTAGTAGCG |

*Cx43, Connexin 43; Cybrd1, Cytochrome b reductase 1; Dmp1, Dentin matrix acidic phosphoprotein 1; Dmt1, Divalent metal transporter 1; Fgf23, Fibroblast growth factor 23; Fth1, Ferritin 1; Slc40a1, Ferropontin-1; Sost, Sclerostin; Tf, Transferrin; TfRc, Transferrin receptor; Tnfsf11, RANKL*
